# Integrative analysis of extracellular and intracellular bladder cancer cell line proteome with transcriptome: improving coverage and validity of –omics findings

**DOI:** 10.1038/srep25619

**Published:** 2016-05-11

**Authors:** Agnieszka Latosinska, Manousos Makridakis, Maria Frantzi, Daniel M. Borràs, Bart Janssen, William Mullen, Jerome Zoidakis, Axel S. Merseburger, Vera Jankowski, Harald Mischak, Antonia Vlahou

**Affiliations:** 1 Biotechnology Division, Biomedical Research Foundation of the Academy of Athens, Athens, Greece; 2 Charité-Universitätsmedizin Berlin, Berlin, Germany; 3 Mosaiques Diagnostics GmbH, Hannover, Germany; 4 GenomeScan B.V., Leiden, The Netherlands; 5 Institut National de la Santé et de la Recherche Médicale (INSERM), Institut of Cardiovascular and Metabolic Disease, Toulouse, France; 6 Université Toulouse III Paul-Sabatier, Toulouse, France; 7 BHF Glasgow Cardiovascular Research Centre, University of Glasgow, Glasgow, United Kingdom; 8 Department of Urology, University of Lübeck, Lübeck, Germany; 9 Department of Urology and Urological Oncology, Hannover Medical School, Hannover, Germany; 10 RWTH-Aachen, Institute for Molecular Cardiovascular Research (IMCAR), Aachen, Germany

## Abstract

Characterization of disease-associated proteins improves our understanding of disease pathophysiology. Obtaining a comprehensive coverage of the proteome is challenging, mainly due to limited statistical power and an inability to verify hundreds of putative biomarkers. In an effort to address these issues, we investigated the value of parallel analysis of compartment-specific proteomes with an assessment of findings by cross-strategy and cross-omics (proteomics-transcriptomics) agreement. The validity of the individual datasets and of a “verified” dataset based on cross-strategy/omics agreement was defined following their comparison with published literature. The proteomic analysis of the cell extract, Endoplasmic Reticulum/Golgi apparatus and conditioned medium of T24 vs. its metastatic subclone T24M bladder cancer cells allowed the identification of 253, 217 and 256 significant changes, respectively. Integration of these findings with transcriptomics resulted in 253 “verified” proteins based on the agreement of at least 2 strategies. This approach revealed findings of higher validity, as supported by a higher level of agreement in the literature data than those of individual datasets. As an example, the coverage and shortlisting of targets in the IL-8 signalling pathway are discussed. Collectively, an integrative analysis appears a safer way to evaluate -omics datasets and ultimately generate models from valid observations.

High-resolution –omics technologies hold the promise of significantly improving our knowledge of disease pathophysiology. Integration of –omics data and their in-depth interpretation in the context of the existing literature, are required to maximize the knowledge extracted from individual datasets. Implementation of this approach could catalyze the development of novel biology-driven drug targets^1^. In particular, studies at the protein level are highly relevant, since proteins directly reflect the disease related phenotypic changes and comprise the vast majority of approved drug targets^2^,^3^. Although recent advances in mass spectrometry (MS)-based technologies enable proteomics investigations with increased sensitivity, numerous challenges remain to be met, mainly related to the proteome vast complexity and (biological) variability, mandating the analysis of multiple independent samples in order to reach statistical significance^4^,^5^. Additionally, to increase proteome coverage, extensive fractionation at the peptide and/ or protein level have been advocated^6^,^7^,^8^. The latter include enrichment strategies for secreted proteins, which have gained increasing attention, as means to understand cancer invasion^9^,^10^,^11^.

Regardless the applied technique, proteomics analysis generally delivers numerous potentially disease-associated proteins. This is especially of value in Systems Biology approaches^12^,^13^,^14^,^15^,^16^ targeting to obtain a spherical view of the disease molecular profile and underlying causative events, and where comprehensiveness is needed. However, verifying all of the identified changes at a single protein level, e.g. via immunohistochemistry or ELISA, appears an impossible task, hence frequently compromising validity of the vast majority of reported –omics findings. To increase confidence on the results obtained from large-scale experiments, an integration of various –omics datasets appears to be a valuable alternative^17^,^18^. In the study presented here, we investigated if cross-omics comparisons and respective investigation of consistency in trends of expression are in fact increasing the validity of the obtained results. In addition, and specifically for proteomics investigations, we target to show that the application of different fractionation strategies, - besides increasing confidence in individual findings via cross-strategy agreement,- increases proteome coverage and facilitates shortlisting of biologically relevant biomarkers.

As a model system, we chose metastatic bladder cancer (BCa) represented by two syngeneic cell lines, T24 and its metastatic subclone T24M. Metastatic BCa is associated with very low survival^19^, hence, understanding the molecular processes and identifying improved therapeutic targets is an unmet, clinical need. High-resolution LC-MS/MS analysis was conducted on samples enriched in secreted proteins, (isolated from conditioned medium-CM and Endoplasmic reticulum and Golgi apparatus (ER/Golgi) fractions, as carrying the cargo of secreted proteins), as well as total cell extract (CE). Total RNA sequencing analysis was utilized to complement and validate the large scale proteomic data sets. To assess the validity of findings in an unbiased way, these findings were compared to literature data represented by the BcCluster BCa database (http://bccluster.org/)^20^ and retrieved using the GLAD4U tool (http://bioinfo.vanderbilt.edu/glad4u/)^21^. As shown, cross-strategy and –omics comparisons at the individual molecule and pathway levels increase the credibility of individual observations and improve proteome coverage consequently increasing data extraction from individual –omics experiments for further systems biology and/or targeted investigation.

## Results

### Proteomic data assessment

**Figure 1:**
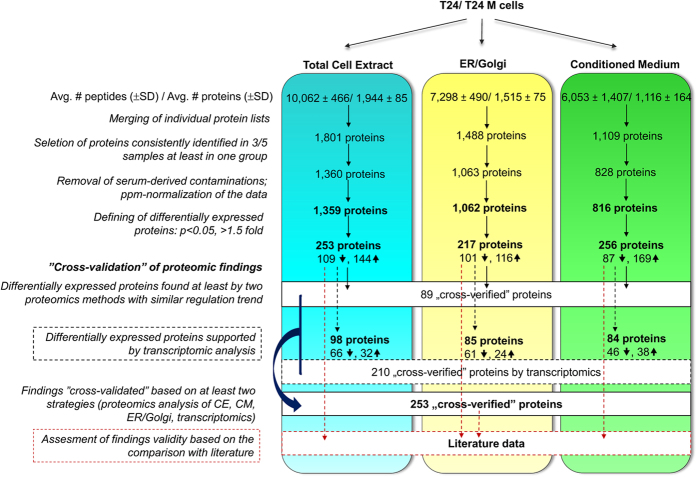
Overview of the study workflow. The main steps of data collection and analysis are presented.

The high-resolution proteomic analysis was performed on samples enriched in secreted proteins (analysis of CM and ER/Golgi fractions) and CE, aiming at increasing proteome coverage. The respective workflow is depicted in Fig. 1. The results from 5 independent experiments per cell compartment indicate high-resolution and good reproducibility of the applied procedures. As shown in Table 1, for each experimental approach an average ( ± SD) of 10,062 ( ± 466), 7,298 ( ± 490), 6,053 ( ± 1,407) peptides, corresponding to 1,944 ( ± 85), 1,515 ( ± 75), 1,116 ( ± 164) proteins were identified in CE, ER/Golgi and CM, respectively. Detailed lists of proteins identified per individual MS-run (including/ excluding single peptide IDs) are provided in Supplementary Table S1. To increase reliability of protein identification and differential expression analysis, only proteins identified based on at least 2 unique peptides (in each individual run) and in at least 3/5 replicates in each cell line were considered for further analysis. The reproducibility rates were high with overlap among replicates on average of 77% (CE), 73% (ER/Golgi) and 76% (CM) of proteins detected in at least 3/5 replicates in each case (Supplementary Fig. S1). These corresponded to a total number of 1,359, 1,062 and 816 non-redundant proteins from CE, ER/Golgi and CM, respectively, considered for further differential expression analysis (Supplementary Dataset S1).

To obtain an estimate of the enrichment efficiency for secreted proteins, the SignalP algorithm, which predicts the presence of signal peptides (defining “secreted” proteins), was employed^22^ (Supplementary Dataset S1). In overall, 30% of proteins in CM were predicted to have signal peptide in comparison to 14% in the ER/Golgi fraction and 9% in CE, indicating the relative efficiency of the enrichment strategies. The normalized signal intensity of these ‘secreted’ proteins corresponded on average to 49.80%, 16.72% and 6.67% of the total intensity for CM, ER/Golgi and CE, respectively. Moreover, enrichment efficiency was assessed based on the normalized average intensity values of specific proteins being representative for each fraction (Supplementary Fig. S2). Actin cytoplasmic 1 and histone H2B type 1-K (protein markers for CE) were highly expressed in CE, whereas their abundance was reduced in CM (by approximately 2 fold for Actin, 10 fold for H2B type 1-K), and for H2B type 1K also reduced (approximately 4 fold) in ER/Golgi (Supplementary Fig. S2). On a similar note, calumenin and 78kDa glucose regulated protein (markers for ER/Golgi) levels were higher in ER/Golgi (by about 2 fold) compared to CE and CM. Cathepsin B and Proactivator polypeptide (markers for CM) levels were found increased (by at least 5 fold) in CM compared to CE and ER/Golgi (Supplementary Fig. S2). Taken together, these results support that the different strategies provided to some extent complementary information. However, large overlaps could also be observed (described below) allowing for investigation of consistencies among the differentially expressed proteins per method, as a means to increase confidence in individual observations.

### Complementarity of proteomic profiles

**Figure 2:**
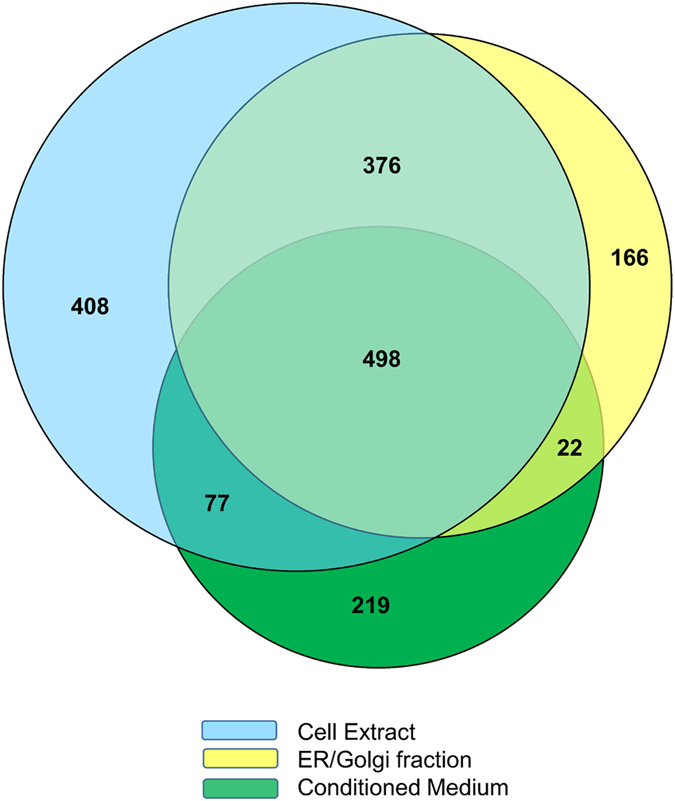
Overview of the numbers of proteins identified in total cell extract, ER/Golgi fraction and conditioned medium. Venn diagram representing a comparative analysis of all proteins (≥2 peptides) identified following application of the three individual protein fractionation strategies.

Comparative analysis of the 1,359 proteins identified in the CE, the 1,062 proteins detected in ER/Golgi and the 816 proteins found in CM revealed an overlap of 498 proteins (Fig. 2). This “core proteome” included multiple enzymes, ribosomal and cytoskeletal proteins, some signalling proteins and also abundant chromosomal proteins (such as histones; Supplementary Dataset S1). Each experimental approach also enabled the identification of multiple proteins not detected by the other two methods (408 for CE, 166 for ER/Golgi and 219 for CM; Fig. 2). The former included various nuclear and transcription factors and mitochondrial enzymes, the ER/Golgi fraction had multiple proteins synthesis-related (Protein Niban, ribosomal proteins, DnaJ homolog subfamily C member etc) and signalling proteins (RAS-related proteins, kinases, cell membrane receptors such as EGFR etc) and the CM fraction included various growth factors, interleukins, matricellular proteins and proteases, indicating a good degree of complementarity between these strategies (Supplementary Dataset S1).

Proteins exhibiting a nominal significant change (p < 0.05, Mann Whitney test) in their expression levels (>1.5 fold change) between the two cell lines in respective subcellular fractions were defined as differentially expressed. Based on these requirements, 253 (144-up and 109-down regulated), 217 (116-up and 101-down regulated) and 256 (169-up and 87-down regulated) proteins were considered as significantly altered among CE, ER/Golgi and CM, respectively in T24M vs. T24 cells (Supplementary Dataset S1). Upon Benjamini-Hochberg correction and considering the adjusted p-value (p < 0.05) and the fold change threshold ( >1.5), a total of 171 and 206 proteins were defined as differentially expressed in CE and CM, respectively (Supplementary Dataset S1); whereas none of the ER/Golgi differentially expressed proteins remained significant upon application of FDR correction. This indicates higher variability of this specific dataset, likely being a consequence of the applied multi-step enrichment protocol. Considering the low number of samples analyzed (n = 5 per group) as well as the observed consistency in expression trends among different fractions (as explained below), we further focused on the differentially expressed proteins (>1.5 fold change) defined using unadjusted p-value.

**Figure 3:**
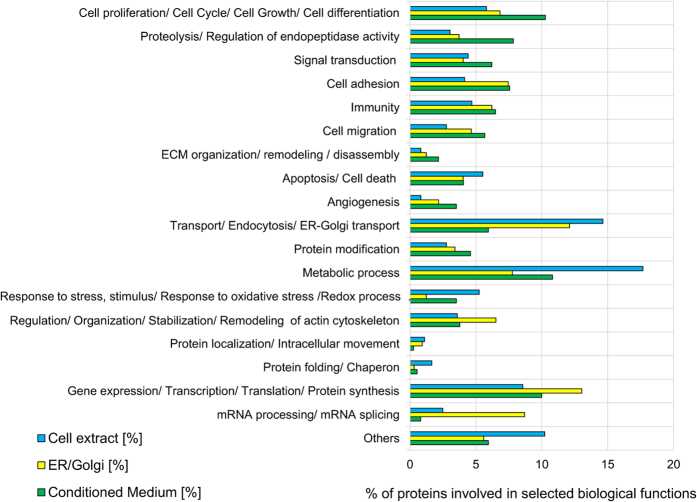
Functional analysis of differentially expressed proteins. Comparison of biological functions for the differentially expressed proteins identified in total cell extract, ER/Golgi fraction and conditioned medium.

To obtain an initial insight in the biological function of the observed differentially abundant proteins per approach (i.e. the aforementioned 253, 217 and 256 proteins identified in CE, ER/Golgi fraction and CM analysis, respectively), gene ontology information deposited in protein databases (Uniprot^23^,^24^ and NeXtProt^25^) was investigated. Comparative analysis revealed that the percentage of differentially expressed proteins involved in metabolic processes, intracellular transport of various compounds (e.g. proteins, ions, lipids), protein folding, redox reactions and response to stress was higher in CE than in CM and ER/Golgi fraction (Fig. 3); whereas differentially expressed proteins implicated in proteolytic events, regulation of endopeptidase activity, extracellular matrix organization/ remodelling, migration, angiogenesis as well as signal transduction and cell proliferation were more prominent in CM vs. CE and ER/Golgi. In addition, the percentage of differentially abundant proteins associated with mRNA processing and splicing, protein synthesis as well as organization of actin cytoskeleton was increased in ER/Golgi when compared to the other samples (Fig. 3). These findings further indicate the complementarity of the applied enrichment strategies.

Consolidation of the differentially abundant proteins from all experimental approaches (CE, ER/Golgi and CM) resulted in a total of 614 non-redundant changes (Supplementary Dataset S2). Some proteins (n = 19) were predicted by all 3 proteomics strategies to be differentially expressed and at similar trends of expression (up or down) in the T24M vs. T24 cells (Table 2). These included proteins involved in actin binding such as gelsolin and plastin-3, proteases (cytosol aminopeptidase), but also various enzymes [Glucose-6-phosphate 1-dehydrogenase, NAD(P)H dehydrogenase [quinone] 1, phospholipase D3, and others]. An additional 70 proteins belonging to various proteins families [signaling molecules such as Signal transducer and activator of transcription 1-alpha/beta; metabolic enzymes such as Fatty acid synthase, UDP-glucose 6-dehydrogenase, Aldehyde dehydrogenase, and others; proteins involved in cell interactions such as Annexins (ANXA2 and ANXA6) etc.] were found to be de-regulated and at similar trends of expression by two fractionation strategies. Collectively, agreement in trends of expression and statistical significance between the strategies increases the confidence in individual observations (a total of 89 differentially expressed proteins as supported by at least two fractionation strategies may be considered “cross-validated”, hence of higher confidence). In addition, and as shown in Supplementary Dataset S2, some significant changes supported by one fractionation strategy were also suggested by other strategies and at the same trends of expression in the T24M versus T24 cells, nevertheless did not pass the applied thresholds (of at least 1.5 fold change and/or p < 0.05) in the latter. This observation (applying to approximately 40 proteins per strategy) further facilitates prioritization and establishing confidence in individual findings.

### Assessment of the validity of proteomic findings by mRNA sequencing analysis

To further assess the validity of the observed proteomic changes, mRNA sequencing data were obtained from the studied cell lines using different biological replicates. Corresponding transcripts for the vast majority of proteins existed. Specifically, corresponding mRNA sequences were found for 1,358 out of 1,359 proteins detected in CE (>99%); 1,061 out of 1,062 proteins in ER/Golgi; and 811 out of 816 proteins detected in CM (>99%).

Among the 253 differentially expressed proteins detected in CE, 98 were also detected with a fold change above 1.5 at the mRNA level. Of the 217 differentially abundant proteins from the ER/Golgi fraction, 85 were also found to be changed at the mRNA levels (fold change >1.5); while among the 256 differentially abundant proteins obtained in CM, 84 were also found to be differentially expressed at the mRNA level. When combined, a total of 210 proteomic changes can be considered as “verified” via agreement with the transcriptomics results (Supplementary Dataset S2). These 210 “verified” findings included various proteins which were defined as differentially expressed in at least two proteomic experiments (Supplementary Dataset S2; proteins marked in red or blue with asterisk) and also proteins which were predicted to be differentially expressed by one only proteomic approach (CE or ER/Golgi or CM; Supplementary Dataset S2; protein marked in green with asterisk), increasing the total number of “verified” findings based on data cross-validation from 89 (cross-validation based on agreement of at least two protein fractionation strategies) to 253 (agreement of at least two –omics strategies; any of protein fractionation approaches and/or transcriptomics, Supplementary Dataset S2). These “verified” features represent a variety of protein families including multiple signalling molecules (e.g. protein kinase C and casein kinase substrate in neurons protein 2, RAS suppressor protein 1, Tyrosine-protein kinase Yes, Interleukin-8, Macrophage colony-stimulating factor 1, Interleukin-6, Vascular endothelial growth factor C and others), proteases (Cathepsin L1, Cytosol aminopeptidase, Carboxypeptidase A4 and others), components of extracellular matrix (such as Fibronectin type III domain-containing protein 3B, Collagen alpha-1(XVIII or XII) chain, Laminin subunit gamma-1 or beta-1, Metalloproteinase inhibitor 3, and others) and also various enzymes (such as NAD(P)H dehydrogenase [quinone]1, Thymidine phosphorylase, Glucose-6-phosphate 1-dehydrogenase, NADH-cytochrome b5 reductase 3 and others).

### Assessment of the validity of the multi –omics approach and its potential application

The validity of the differentially expressed proteins reported in individual proteomics experiments (CE, ER/Golgi, CM) as well as in the integrated “verified” dataset (abovementioned 253 proteins) was evaluated in the context of existing literature. Molecular features associated with BCa invasion or metastasis were retrieved using two independent approaches i.e. the BcCluster database^20^ (n = 627) and the GLAD4U^21^ tool (n = 671; Supplementary Dataset S3), as described in Methods. Validity was assessed based on the overlap between our experimental data and literature findings (listed in Supplementary Dataset S3). As presented in Table 3, the percentage of overlapping features between the “compiled” (CE, CM, ER/Golgi, i.e. 614 proteins) dataset and literature, was 8.8% (BcCluster) and 11.6% (GLAD4U); whereas for the verified findings (i.e. 253 proteins), the agreement with the literature data was generally higher (overlap range: 13.0–15.8% depending on the comparison (Table 3).

**Figure 4:**
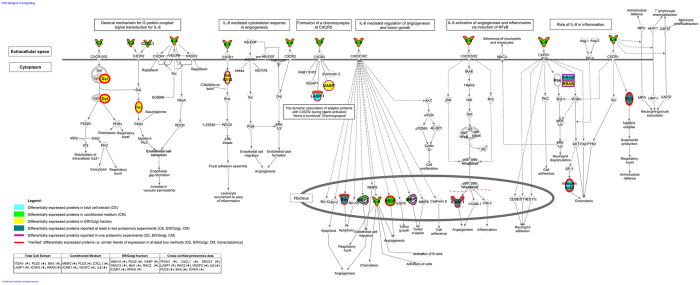
Graphical representation of the IL-8 signaling pathway based on multi-omics profiling. Protein changes identified by each of the individual proteomics strategies (CE, ER/Golgi, CM) are shown, as well as those supported by at least two experimental strategies (CE, ER/Golgi, CM, transcriptomics). The expression trend of each molecule in T24M vs. T24 cells is indicated with arrows (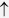 for up- and 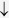 for down-regulated proteins in T24M vs. T24).

Considering the increased validity of the latter dataset, these 253 proteins were mapped to pathways using the Ingenuity software. The predicted statistically significant de-regulated pathways (p < 0.05, Fisher exact test) were shortlisted, based on their significance level, and the top 15 pathways with the lowest p-value are summarized in Table 4. As a representative example, we present the IL-8 signalling pathway, which notably was the only one found in the top 15 significant pathways predicted based on the literature data and also significant on each individual proteomics dataset. The graphical representation of the IL-8 signalling pathway is shown on Fig. 4 with the differentially expressed features, as detected per individual –omics method, highlighted. As presented, the molecular coverage of the IL-8 pathway increases through the integrative analysis, further reflecting the complementarity of the different approaches. Furthermore, as shown, the vast majority of molecular changes are considered “verified” (Fig. 4 - red frame). Based on this scheme, the chances that the observed “non-verified” changes (e.g. changes supported by one only –omics approach; purple frame) are valid increase, based on their biological relevance. To test this hypothesis the differential expression of the Vasodilator-stimulated phosphoprotein (VASP) was investigated in a set of invasive and non-invasive BCa tissue specimens by western blot. As shown in Supplementary Fig. S3, in line with the ER/Golgi proteomics analysis, a decrease in the level of this protein in invasive versus non-invasive tumors is suggested.

## Discussion

Omics datasets are mine of information, nevertheless only a limited part of it is finally extracted and further investigated mainly due to challenges associated with: a) establishing reliability of findings (typically large numbers of differentially abundant proteins of low statistical power are identified per omics experiment); and b) developing targeted assays for further measurement of individual features, as a means for their verification. Particularly, frequent lack of specific antibodies and the associated costs of performing immuno-based assays result in only a small number of proteomics findings being ultimately confirmed (typically less than 10 per experiment). These verified findings, even though of high value, are not sufficient to comprehensively describe a disease at the molecular level. However, such comprehensive description is required for the successful application of spherical “systems biology” approaches^12^. In the case of proteomics studies more specifically, comprehensiveness and proteome coverage are dependent on the applied technique, with different subcellular fractions requiring the use of different enrichment strategies for their efficient resolution. The presented approach involving use of different enrichment strategies as well as transcriptomics, addressed the added value of cross-strategy, cross-omics comparisons and respective investigation of consistency in trends of expression, in increasing confidence in individual findings per omics dataset.

We focused on the analysis of BCa metastasis using a cell line model for the specific phenotype. This constitutes a clinically relevant question, as limited therapeutic options are available for patients with BCa metastatic disease, highlighting the need for development of novel therapeutic targets^19^.

We placed special emphasis on the investigation of the secreted/ extracellular matrix proteome, considered of high relevance in cancer invasion and metastasis^11^. In parallel to the classical analysis of CM, we also investigated the ER/Golgi fraction, representing the path of proteins on their way to be secreted^26^. As recently demonstrated and also shown in our analysis, this latter method is of lower enrichment efficiency for bona fide secreted proteins in comparison to the analysis of CM, nevertheless, it can provide new information and highly complementary results to the latter (CM)^27^. Even though one cannot rule out the possibility that some of the observed differences (or overlaps) between protein identifications from different fractions may reflect sub-optimal enrichment and/or differences in starting protein amounts (e.g. 5 μg for CE, 3.5 μg for ER/Golgi and 3.75 μg for CM analyzed by LC-MS/MS - Methods), the overall specificity of the employed techniques is supported by the Signal IP analysis and respective analysis of protein abundance per fraction (Supplementary Fig. S2). Furthermore, the overall enrichment efficiency in our study is in line with previously published reports^28^,^29^.

Several proteomics studies have been published involving characterization of changes underlying BCa invasion either at the total cell^30^,^31^,^32^ or extracellular proteome^33^,^34^,^35^ using various BCa cell line models. A high overlap between the reported identifications in our study and the existing literature was observed, further supporting the validity of the reported findings. Specifically, our shotgun analysis enabled detection of the majority (at least 69%) of proteins identified in previous proteomics studies of total cell proteome from T24M vs. T24^31^ and T24T vs. T24^30^ cells. Along the same lines, the majority of proteins previously identified in CM from T24M and/or T24 cells^33^,^35^ were also found in our analysis. These multiple existing studies serve as reference points, nevertheless their findings remain disparate and any potential added value from the parallel proteomic analysis of different cell compartments can be assessed with moderate confidence only.

As the first step in this direction, and to establish the relevance of each individual proteomics dataset, we evaluated our main findings in the context of the existing literature. We used a manually curated database of features (genes, transcripts, proteins) associated with BCa invasion/ progression (BcCluster)^20^. Importantly, BcCluster lists molecules highlighted from studies with sample size of at least 50, suggesting a high validity of the collected features. The second dataset contains the list of the BCa-associated molecules retrieved using the GLAD4U^21^ tool, without any sample size selection criteria. The two datasets appear to be highly complementary, with an overlap of 179 features, (corresponding to over 25% of features from each literature set), further supporting the assumption that these two approaches provide a good and comprehensive reflection of the current knowledge. It should be noted, that these literature data used as reference in our study include entries reported from different –omics (genomics, proteomics, transcriptomics) as well as non-omics (e.g. immunohistochemistry) studies, apparently collected under different applied methodologies. Investigation of the inter-laboratory variability reflected in these databases would be out of the realm of this study, nevertheless it is expected that this exists. Even though the latter clearly compromises comparability of different studies, on a positive note, it may also be used as a means to increase confidence in individual findings, based on their detection in multiple studies and under different protocols. Along these lines, multiple observed protein changes included in the individual datasets (CE, ER/Golgi, CM) had already been reported in the context of BCa invasion/ progression or metastasis. Independently of the source of literature data, as aforementioned (Results), overlap ranged from approximately 7 to 15% depending on the applied proteomics strategy.

Taking this one step further, an integration of –omics datasets from different molecular levels (proteomics - transcriptomics) was also performed. For almost all proteins identified by LC-MS/MS, we were able to obtain the corresponding mRNA, which strongly supports the reliability of the protein identification process. Even though, in general a moderate correlation between mRNA and protein expression is reported^18^,^36^, the regulation trend was well supported by the transcriptomic analysis for many of the differentially expressed proteins (210 out of 614 (34%); notably for 344 transcripts a >1.5-fold change was not reached, whereas only 57 exhibited opposite trend of expression in the T24M vs T24 cells). It should be noted that the presented transcriptomic analysis has some limitations, mostly as a result of the high costs of the next generation sequencing analysis, resulting in a low number of analysed samples (n = 2 per cell line) hampering the application of proper statistical analysis.

Through the application of transcriptomics, which complements but also verifies proteomics findings, an increase in the number (from 89 up to 253) of cross-validated features obtained in the three individual proteomics experiments could be achieved.

The reliability of these latter “cross-validated” proteins was further evaluated in the context of available literature. An improvement in the agreement with existing literature data is observed (as described in Table 3), indicating the applicability and value of such a multi-omics approach to verify large scale proteomics data. Of these 253 features, 33 (13.0% BcCluster)^20^ or 40 (15.8% Glad4U)^21^ have been associated with BCa/ BCa invasion or metastasis. This corresponds to approximately a 5% increase in the overlapping features when compared with the respective overlap of all 614 differentially abundant proteins, identified across the three proteomics experiments. This increase appears to be significant, considering that the “verified” dataset consists of a lower number of proteins (253) compared to the combined “all differentially expressed proteins” dataset (614). In other words, the presented strategy facilitates shortlisting more confident findings, which currently range from the small number (regularly less than 10) of verified findings via typical targeted analysis, to the whole list of differentially expressed features per omics experiment (regularly prone to many false positives). The described cross-omics comparison offers the valuable intermediate step between these two extremes, allowing to maximize extraction of features of increased confidence for their further use as input data in systems biology approaches.

As an example in this direction, pathway analysis was conducted. IL-8 signaling was selected, as being predicted (at high significance levels) to be affected based on all, literature mined datasets as well as individual proteomics datasets (CE, ER/Golgi and CM). As presented in Fig. 4, the integrative analysis of –omics data provided a fairly comprehensive molecular phenotype underlying the pleiotropic effects of IL-8 function: The up-regulation of IL-8 in the T24M cells was associated with an up-regulation of matrix metalloproteases (MMP2), implicated in tumor invasion^37^, as well as VEGFC and ICAM1, factors implicated in angiogenesis^38^,^39^ (Fig. 4). Interestingly, the overexpression of MMP2 was accompanied by the down regulation of TIMP metallopeptidase inhibitor 3 (identified in CM analysis), further supporting the activation of MMPs in the context of BCa invasion. Even more: data integration from the different preparation methods (CE, ER/Golgi, CM) links disparate observations revealing events in cases not associated with BCa yet. As shown on Fig. 4, formation of chemosynapse is predicted based on the observed proteomics changes (involvement of VASP, LASP-1), with anticipated impact on focal adhesion and cell migration^40^,^41^. In addition, interestingly, involvement of PLD3, a non-classical member (as it lacks lipase activity) of the phospholipase D family of enzymes^42^ is predicted. PLD enzymes have been implicated as key components of HRAS signaling in cancer cells^43^ –with, notably, HRAS also detected at different levels in T24M versus T24 cells, based on the proteomics analysis (Fig. 4). In addition, PLD3 has been recently shown to be involved in hypoxia-induced lipid metabolism in colorectal cancer cells^44^, suggesting collectively, that it merits further investigation in BCa. In parallel to these effects, IL-8 signaling also occurs through G protein coupled receptors, specifically in our system, through Guanine nucleotide-binding protein subunit gamma-12 (GNG12), not studied in BCa yet. Impacts on regulation of calcium channels are expected^45^,^46^. Of note, some calcium channels were found at differential levels in the T24M versus T24 cells based on the proteomics analysis e.g. Plasma membrane calcium-transporting ATPase 1 (CE and ER/Golgi), Calcium-binding mitochondrial carrier protein SCaMC-1 (CE)-Supplementary Dataset S2.

Collectively, through the proposed combined analysis of multiple cellular fractions and molecular levels, these multi-level pleiotropic effects of IL-8 previously described in different publications (reviewed by Waugh *et al*)^47^ can be better reflected at the molecular level, encompassing changes at the extracellular space (e.g. IL-8 differential abundance), all the way to the nucleus (e.g. changes on Bax; Fig. 4). There is no doubt that multiple missing links still exist nevertheless, such an approach obviously increases coverage (hence confidence), but also facilitates definition of targets for further verification. To better explain this point, the example of the VASP, a protein involved in cytoskeleton remodeling^41^ and not yet associated with BCa was provided. Being differentially expressed in the ER/Golgi fraction (only), VASP was not included in the shortlisted proteins (i.e. the 253 cross-verified findings). Nevertheless, based on its biological relevance to the IL-8 pathway, the chances that this finding from the ER/Golgi analysis was not a false association increased. Indeed, by using western blot analysis, our preliminary results further supported the down-regulation of VASP in muscle invasive BCa, a finding which we currently further investigate.

In conclusion, our study collectively shows that comparative and in parallel analysis of multiple –omics (in our case: proteins identified in CE, ER/Golgi and CM and also at a different omics level - transcriptomics) has added value on two very important aspects; it can improve proteome coverage and fill missing links, through the complementarity of different techniques. Even more, it can increase validity of individual observations, by cross-omics correlations, facilitating prioritization of findings and ultimately knowledge extraction. Considering the general low statistical power of individual –omics investigations (high number of variables, small sample sizes) such a cross-omics and platform analysis appears a safe way forward particularly towards development of disease molecular models based on valid experimental observations.

## Methods

### Sample preparation

#### Cell culture

T24 and T24M^31^ BCa cells were employed as described in Makridakis *et al*^31^. Briefly, cells were cultivated in DMEM medium (High Glucose, GlutaMAX™, Pyruvate) supplemented with 10% FBS and 1% Penicillin-Streptomycin (P/S) and harvested using 0.05% trypsin/0.02% EDTA and centrifugation (1,000× g, 5 min, room temperature). Cell pellets were washed twice with PBS and stored at −80 °C until further processing. Each experiment was repeated in five replicates (five different flasks with cells originated from same initial stock) per condition.

### Collection of secreted proteins from conditioned medium (CM)

CM was collected are described previously^27^,^35^ from 10∙10^6^ cells after 24h incubation in serum deprived medium. Protein extraction was performed as described in Latosinska *et al*^27^. 75 μg of proteins were processed by Filter Aided Sample Preparation method (FASP), as described below.

### Enrichment in Endoplasmic Reticulum/ Golgi Fraction

20∙10^6^ cells were used in order to enrich for ER/Golgi as described by Sarkar *el al*.^26^ with minor modifications^27^. Sequentially, samples were depleted in nuclei (3,000 × g, 10 min) and mitochondria (10,000 × g for 10 min) leading to enrichment for ER/Golgi (16,000 × g for 30 min). Pellet containing the final fraction was dissolved in buffer containing 7M urea, 2M thiourea, 4% CHAPS, 100 mM DTE and 1% ampholytes. 70 μg of proteins were processed by FASP.

### Preparation of total cell extract

4∙10^6^ cells were harvested and cell pellet was re-suspended in 200 μL of lysis buffer (7M urea, 2M thiourea, 4% CHAPS, 100 mM DTE, 1% ampholytes). Cells were disrupted by water bath sonication for 10 min followed by centrifugation (16,000× g, 10 min, RT). 100 μg of proteins were processed by FASP.

### Filter aided sample preparation (FASP)

FASP was performed according to Wisniewski *et al*^48^ with minor modifications^49^. Briefly, sequential buffer exchange with urea buffer and ammonium bicarbonate (after alkylation with 100 μL of 0.05M IAA) was performed in Amicon Ultra Centrifugal filter devices (0.5 mL, 30 kDa MWCO, Millipore) at 16,000× g for 15 min at room temperature. Proteins were digested overnight on filters with 1:100 trypsin to protein ratio. Centrifugation and lyophilisation were then applied^49^.

### LC-MS/MS analysis

Lyophilized peptides were re-dissolved in 100 μL of HPLC grade water. Subsequently, 5 μL of the peptide mixture was analysed on a nano-flow system (Dionex Ultimate 3000 RSLS, Dionex, Camberley UK), as described before^27^. Briefly, samples were loaded onto a Dionex nano trap column (C18, 0.1 × 20 mm 5 μm) at a flow rate of 5 μl/min in 98% 0.1% formic acid and 2% acetonitrile, followed by elution onto an Acclaim PepMap nano column (C18, 75 μm × 50 cm, 2 μm 100 Å) at a flow rate of 0.3 μl/min. Reverse-phase chromatography was performed using a linear gradient of solution A [0.1% formic acid and acetonitrile (98:2)] and solution B [0.1% formic acid and acetonitrile (20:80)]. Separation was initiated using 1% solution B (5 min) followed by a gradual increase to 30% (400 min) and 50% (480 min). Ionization involved a nano electrospray source (Proxeon, Thermo Fisher Hemel UK) in a positive ion mode and MS/MS an Orbitrap Velos FTMS (Thermo Finnigan, Bremen, Germany). Ionization voltage was 2.6 kV and the capillary temperature was 200 °C. The mass spectrometer was operated in MS/MS mode scanning from 380–2,000 amu. The MS analysis was performed using a data-dependent acquisition (top 40). Changes between MS1 (MS) and MS2 (MS/MS) modes were done at 60,000 and 7,500 resolution respectively. Parent ions were fragmented at and energy of 40 by higher energy collision-induced dissociation (HCD).

### Data processing

The analysis of the raw MS data files was performed using Proteome Discoverer (PD) v. 1.4.0.288 (Thermo Scientific). An event detection node was used at a setting of 2 ppm. The Human Swiss-Prot Database^24^,^50^ with 20 277 canonical sequences only (downloaded at 30/10/2013) and the Sequest search engine^51^ were employed. The following criteria were applied: a) precursor mass tolerance 10 ppm, b) fragment mass tolerance 0.05 Da, c) fix modifications: carbamidomethylation of cysteine, d) variable modifications: oxidation of methionine and proline, and e) allowed missed cleavages: one. The false discovery rate (FDR) evaluation was performed by using the Percolator node^52^ (PD 1.4).

### Protein identification and label-free quantification

Protein identification was based on the rank 1 peptides allowing for mass deviation below 5 ppm and FDR below 1%. Only proteins identified with at least 2 unique peptides in individual samples were included for further analysis. The label-free quantification was based on the peak area (i.e. area under the curve), determined based on the extracted ion chromatogram (Precursor Ions Area Detector node in PD). Quantification at the protein level was based on the top three peptides per protein calculated by PD. For the few cases where the protein area was not calculated by the software, as a consequence of lack of integration of the peptide area (a software error), the average area for the particular protein per studied group (T24, T24M) was assigned. In the case of proteins not identified in a particular sample, the missing value was replaced by zero. Twelve proteins derived from the FBS^53^ or reagents used for MS were excluded from analysis as potential contaminations (Supplementary Table S2). A part per million (ppm)-normalization was conducted as follows: 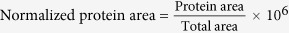 (equation 1), where the total area was defined as a sum of protein areas in each sample. Statistics was performed using SPSS Statistics 17.0 (Mann-Whithney U Test) and R-Package (Benjamini-Hochberg correction).

### Total mRNA sequencing

Total RNA from T24 and T24M cells was isolated from 10∙10^6^ cells by TRI Reagent (Sigma Aldrich) (2 replicates per condition) and obtained RNA extracts were purified with RNeasy cleanup kit (Qiagen) including prior digestion with DNase I; both steps were performed according to manufacturer’s protocol. The preparation of libraries and sequencing of the mRNA along with the analysis of the raw data was performed by GenomeScan B.V. The RNA concentration was assessed using the Life Technologies Qubit. Further evaluation of the quality and integrity of isolated RNA was conducted using Agilent Bioanalyzer. Subsequently, samples were processed by Illumina^®^ mRNA-Seq Sample Prep Kit according to Illumina protocol. Briefly, mRNA isolation was performed using oligo-dT magnetic beads followed by mRNA fragmentation and cDNA synthesis. For the latter, the quality and yield was measured via Lab-on-a-Chip analysis (expected product size: 200–500 bp). Clustering and DNA sequencing were performed using Illumina cBot and HiSeq2500 in line with manufacturer’s instructions at the concentration of 16pM of DNA. Image analysis, base calling and the quality check were conducted using the Illumina data analysis pipeline RTAv1.18.64 and Bclfastqv1.8.4. Data obtained from the HiSeq2500 in fastq format was used as source for the downstream data analysis. Alignment of fastq reads was performed using TopHat version 2.0.12^54^ against the assembled human genome GRCh37.p13 with the corresponding Ensembl release 75 annotation^55^ (http://grch37.ensembl.org/index.html). The alignment run involved default parameters but allowing for a genome multihit search and transcriptome build and mapping. Alignment quality metrics were collected using Qualimap version 2.0.1^56^. Quantification of feature alignments was performed using HTSeq-counts from HTSeq framework version 0.6.1p1^57^. Default parameters were used for a non stranded RNA-seq library using the intersection non empty algorithm. Normalization of the count data and statistical analysis for the differential expression was performed with DESeq2 package version 1.6.3^58^ for R statistical computing software^59^.

### Western Blot

BCa tissue specimens were collected in Germany (Department of Urology and Urological Oncology, Hannover Medicine School) from patients undergoing resection of the bladder. All individuals gave written informed consent. All experimental protocols for tissue sample collection were approved by the Hannover Medical School Ethics committee (case number: 614–2009) and experiments were performed according to relevant guidelines. Specimens from non-muscle invasive (n = 3), muscle invasive (n = 3) BCa and negative biopsies (n = 3) were analyzed. Tissue lysis was performed as described earlier^49^. 20 μg of total protein per extract were separated by NuPAGE® Gradient Gel 4–12% under reducing conditions and electroblotted to nitrocellulose membrane (LG), as presented elsewhere^60^. Membranes were incubated overnight at 4 °C with the primary mouse anti-VASP antibody (Enzo LifeScience, ALX-804-177-C050, dilution 1:500) or anti- β-actin antibody conjugated to HRP (Santa Cruz, sc-47778 HRP, 1:4,000), in the first case followed by incubation with anti-mouse HRP-conjugated secondary antibody (Santa Cruz; dilution 1:2,000) for 2h at room temperature. Target protein was detected by Enhanced Chemiluminescence (Perkin-Elmer LAS, Inc.).

### Literature mining

Molecules (proteins and transcripts) associated with BCa invasion/ progression were retrieved from the BCa database (http://bccluster.org/)^20^. GLAD4U^21^ was also employed to retrieve relevant featured from MEDLINE database using the following keywords: (“bladder cancer” or “urothelial cancer” or “transitional cell carcinoma” or “urothelial cancer”) and (“invasion” or “progression” or “invasiveness” or “aggressiveness” or “metastasis”) with the undefined threshold settings for genes prioritization.

### Functional annotation

The biological function of the differentially expressed proteins was manually evaluated based on the Gene Ontology (GO) annotations retrieved from Uniprot-GOA annotations^23^ and/ or NeXtProt database^25^. In parallel, differentially expressed proteins which were considered as “verified” were mapped to pathways using QIAGEN’s Ingenuity® Pathway Analysis (IPA®, QIAGEN Redwood City, www.qiagen.com/ingenuity). Statistical analysis was conducted by using right-tailed Fisher’s exact test. Pathways with a p-value below 0.05 were considered as significant.

## Additional Information

**How to cite this article**: Latosinska, A. *et al*. Integrative analysis of extracellular and intracellular bladder cancer cell line proteome with transcriptome: improving coverage and validity of -omics findings. *Sci. Rep.* **6**, 25619; doi: 10.1038/srep25619 (2016).

## Supplementary Material

Supplementary Information

Supplementary Dataset 1

Supplementary Dataset 2

Supplementary Dataset 3

## Figures and Tables

**Table 1:** Summary of the average number of peptides and proteins (including single peptide identifications) identified in individual samples (n = 5) in the T24 and T24M bladder cancer cells.

	Conditioned Medium	ER/Golgi Fraction	Total Cell Extract
**Avg. number of peptides**			
T24	5,814 ± 19,48	7,354 ± 295	9,887 ± 515
T24M	6,292 ± 716	7,241 ± 667	10,237 ± 382
T24 and T24M	6,053 ± 14,07	7,298 ± 490	10,062 ± 466
**Avg. number of proteins**			
T24	1,083 ± 224	1,530 ± 55	1,922 ± 108
T24M	1,150 ± 86	1,501 ± 96	1,965 ± 57
T24 and T24M	1,116 ± 164	1,515 ± 75	1,944 ± 85

**Table 2:** Differentially expressed proteins in T24M versus T24 cells supported by all three proteomics strategies.

Accession	Protein Name	Cell-Extract		ER/Golgi		Conditioned Medium	
		Fold Change	p-value	Fold Change	p-value	Fold Change	p-value
P08582**	Melanotransferrin	only T24M	0.01	2.52	0.01	Only T24M	0.01
P05161**	Ubiquitin-like protein ISG15	4.99	0.01	6.06	0.01	Only T24M	0.01
Q13642**	Four and a half LIM domains protein 1	2.31	0.01	1.86	0.01	10.8	0.01
P61769**	Beta-2-microglobulin	1.78	0.05	16.68	0.03	1.60	0.03
Q14011**	Cold-inducible RNA-binding protein	1.52	0.01	Only T24M	0.02	1.76	0.05
Q09666**	Neuroblast differentiation-associated protein AHNAK	0.64	0.01	0.52	0.01	0.54	0.01
Q01813**	6-phosphofructokinase type C	0.54	0.01	0.30	0.01	only T24	0.02
P15559**	NAD(P)H dehydrogenase [quinone] 1	0.54	0.01	0.51	0.05	0.12	0.01
P06396**	Gelsolin	0.53	0.01	0.51	0.01	0.48	0.01
Q9UBG0**	C-type mannose receptor 2	0.52	0.03	0.4	0.02	0.54	0.01
P11413**	Glucose-6-phosphate 1-dehydrogenase	0.52	0.01	0.51	0.01	0.56	0.01
P08670	Vimentin	only T24M	0.01	Only T24M	0.01	Only T24M	0.01
P28838	Cytosol aminopeptidase	2.22	0.01	3.57	0.01	2.46	0.01
P13797	Plastin-3	1.79	0.01	1.63	0.01	1.66	0.01
P05362	Intercellular adhesion molecule 1	1.64	0.01	2.3	0.01	3.05	0.01
O95336	6-phosphogluconolactonase	1.60	0.01	3.79	0.01	4.15	0.01
P26639	Threonine--tRNA ligase, cytoplasmic	1.54	0.01	1.50	0.01	2.32	0.01
P00492	Hypoxanthine-guanine phosphoribosyltransferase	1.51	0.01	2.12	0.01	1.77	0.01
Q8IV08	Phospholipase D3	1.86	0.01	Only T24M	0.02	only T24M	0.02

^**^Differential expression (fold change >1.5) was also supported by transcriptomics.

**Table 3:** Assessment of the validity of proteomics findings based on literature.

Cell line study (T24M vs. T24)			# overlapping molecules	
	Category	# molecules	BcCluster	Glad4U
Individual proteomic analysis	Total cell extract	253	19 (7.5%)	27 (10.7%)
	ER/Golgi	217	22 (10.1%)	28 (12.9%)
	Conditioned medium	256	27 (10.5%)	38(14.8%)
	Compilation from all proteomics methods	614	54 (8.8%)	71 (11.6%)
“omics” verified findings	Agreement in all 4 strategies	11	2 (18.2%)	4 (36.4%)
	Agreement in 2 or 3 out of 4 strategies	242	31 (12.8%)	36 (14.9%)
	All verified proteins	253	33 (13.0%)	40 (15.8%)

Findings from individual proteomics experiments (CE, ER/Golgi, CM) as well as from the “verified” dataset (established based on statistical significance and expression trend agreement between at least 2 strategies e.g. proteomics analysis of CE, ER/Golgi, CM, and transcriptomics) were evaluated. Proteins extracted from the bladder cancer database (BcCluster)^20^ and by using GLAD4U^21^ were utilized as reference.

**Table 4:** Top 15 Ingenuity Canonical Pathways predicted to be enriched (p < 0.05) based on the integrated “verified” dataset.

#	Ingenuity Canonical Pathways	Molecules	Rank, Cell extract	Rank, ER/Golgi	Rank, CM	Rank, Glad4U	Rank, BCCluster
1	Interferon Signaling	IFIT1, STAT1, IFIT3, ISG15, MX1, IFI35, BAX	**5**	**3**	Not predicted	*303*	*272*
2	Superoxide Radicals Degradation	SOD2, SOD3, CAT, NQO1	*34*	92 (n.s.)	**8**	*313*	Not predicted
3	Caveolar-mediated Endocytosis Signaling	HLA-A, ITGAV, DNM2, ITGB1, ITGA6, B2M, HLA-B, FLOT2	*28*	**5**	*20*	*282*	*233*
4	Hepatic Fibrosis / Hepatic Stellate Cell Activation	NFKB2, IGFBP4, COL12A1, STAT1, CSF1, BAX, IL6, ICAM1, VEGFC, COL18A1, CXCL8, MYH9	200 (n.s.)	197 (n.s.)	**2**	**4**	**8**
5	Role of Tissue Factor in Cancer	ITGAV, ITGB1, CSF1, ITGA6, CXCL1, YES1, VEGFC, CXCL8, ARRB1	100 (n.s.)	*19*	*34*	*27*	*15*
6	Role of IL-17F in Allergic Inflammatory Airway Diseases	NFKB2, CXCL6, CXCL10, CXCL1, IL6, CXCL8	275 (n.s.)	Not predicted	**11**	*120*	*242*
7	Putrescine Degradation III	ALDH3A2, IL4I1, ALDH3A1, ALDH1A3	*19*	*45*	149 (n.s.)	348 (n.s.)	336 (n.s.)
8	Tryptophan Degradation X (Mammalian, via Tryptamine)	ALDH3A2, IL4I1, ALDH3A1, ALDH1A3	*21*	*47*	154 (n.s.)	351 (n.s.)	340 (n.s.)
9	Ethanol Degradation IV	ALDH3A2, CAT, ALDH3A1, ALDH1A3	**6**	*52*	Not predicted	357 (n.s.)	420 (n.s.)
10	Complement System	CFB, C3, C1QBP, C1S, C1R	Not predicted	195 (n.s.)	**3**	Not predicted	339 (n.s.)
11	Dopamine Degradation	ALDH3A2, IL4I1, ALDH3A1, ALDH1A3	*27*	*59*	168 (n.s.)	*320*	362 (n.s.)
**12**	**IL-8 Signaling**	**ITGAV, PLD3, CXCL1, GNG12, BAX, LASP1, RAC2, ICAM1, VEGFC, CXCL8**	*75*	*17*	*62*	**8**	**4**
13	Virus Entry via Endocytic Pathways	HLA-A, DNM2, ITGB1, ITGA6, B2M, HLA-B, RAC2	*72*	**4**	*24*	*213*	*99*
14	Role of Pattern Recognition Receptors in Recognition of Bacteria and Viruses	EIF2AK2, NFKB2, C3, OAS3, OAS2, DDX58, IL6, CXCL8	*59*	128 (n.s.)	*23*	*55*	*166*
15	Pentose Phosphate Pathway	TKT, G6PD, PGLS	**8**	*26*	*26*	380 (n.s.)	391 (n.s.)

Pathways were ranked based on the significance level. Subsequently, the overlap between the top 15 pathways, as defined based on the integrated “verified” dataset, and pathways predicted based on the individual proteomics datasets (CE, ER/Golgi, CM) and literature mined dataset was established. The respective rank for the overlapping pathways is indicated. Significant pathways with the rank ≤ 15 are marked in bold, while significant pathways with rank > 15 are marked in italics. (n.s. not significant results).

## References

[b1] Tanaka H. Omics-based medicine and systems pathology. A new perspective for personalized and predictive medicine. Methods Inf Med 49, 173–185 (2010). 20177649 10.3414/ME9307

[b2] Imming P., Sinning C. & Meyer A. Drugs, their targets and the nature and number of drug targets. Nat Rev Drug Discov 5, 821–834 (2006). 17016423 10.1038/nrd2132

[b3] Overington J. P., Al-Lazikani B. & Hopkins A. L. How many drug targets are there? Nat Rev Drug Discov 5, 993–996 (2006). 17139284 10.1038/nrd2199

[b4] Rifai N., Gillette M. A. & Carr S. A. Protein biomarker discovery and validation: the long and uncertain path to clinical utility. Nat Biotechnol 24, 971–983 (2006). 16900146 10.1038/nbt1235

[b5] Dakna M. . Addressing the challenge of defining valid proteomic biomarkers and classifiers. BMC Bioinformatics 11, 594 (2010). 21208396 10.1186/1471-2105-11-594 PMC3017845

[b6] Huber L. A., Pfaller K. & Vietor I. Organelle proteomics: implications for subcellular fractionation in proteomics. Circ Res 92, 962–968 (2003). 12750306 10.1161/01.RES.0000071748.48338.25

[b7] Lee Y. H., Tan H. T. & Chung M. C. Subcellular fractionation methods and strategies for proteomics. Proteomics 10, 3935–3956 (2010). 21080488 10.1002/pmic.201000289

[b8] Yates J. R. 3rd, Gilchrist A., Howell K. E. & Bergeron J. J. Proteomics of organelles and large cellular structures. Nat Rev Mol Cell Biol 6, 702–714 (2005). 16231421 10.1038/nrm1711

[b9] Makridakis M. & Vlahou A. Secretome proteomics for discovery of cancer biomarkers. J Proteomics 73, 2291–2305 (2010). 20637910 10.1016/j.jprot.2010.07.001

[b10] Paltridge J. L., Belle L. & Khew-Goodall Y. The secretome in cancer progression. Biochim Biophys Acta 1834, 2233–2241 (2013). 23542208 10.1016/j.bbapap.2013.03.014

[b11] Lu P., Weaver V. M. & Werb Z. The extracellular matrix: a dynamic niche in cancer progression. J Cell Biol 196, 395–406 (2012). 22351925 10.1083/jcb.201102147 PMC3283993

[b12] Ge H., Walhout A. J. & Vidal M. Integrating ‘omic’ information: a bridge between genomics and systems biology. Trends Genet 19, 551–560 (2003). 14550629 10.1016/j.tig.2003.08.009

[b13] Bhat A. . Protein interactome of muscle invasive bladder cancer. PLoS One 10, e0116404 (2015). 25569276 10.1371/journal.pone.0116404 PMC4287622

[b14] Cisek K., Krochmal M., Klein J. & Mischak H. The application of multi-omics and systems biology to identify therapeutic targets in chronic kidney disease. Nephrol Dial Transplant (2015). 10.1093/ndt/gfv364 26487673

[b15] Husi H. . A combinatorial approach of Proteomics and Systems Biology in unravelling the mechanisms of acute kidney injury (AKI): involvement of NMDA receptor GRIN1 in murine AKI. BMC Syst Biol 7, 110 (2013). 24172336 10.1186/1752-0509-7-110 PMC3827826

[b16] Molina F. . Systems biology: opening new avenues in clinical research. Nephrol Dial Transplant 25, 1015–1018 (2010). 20139409 10.1093/ndt/gfq033

[b17] Gry M. . Correlations between RNA and protein expression profiles in 23 human cell lines. BMC Genomics 10, 365 (2009). 19660143 10.1186/1471-2164-10-365 PMC2728742

[b18] Maier T., Guell M. & Serrano L. Correlation of mRNA and protein in complex biological samples. FEBS Lett 583, 3966–3973 (2009). 19850042 10.1016/j.febslet.2009.10.036

[b19] van den Bosch S. & Alfred Witjes J. Long-term cancer-specific survival in patients with high-risk, non-muscle-invasive bladder cancer and tumour progression: a systematic review. Eur Urol 60, 493–500 (2011). 21664041 10.1016/j.eururo.2011.05.045

[b20] Bhat A. . BcCluster: A Bladder Cancer Database at the Molecular Level. Bladder Cancer 2, 65–76 (2016). 10.3233/BLC-150024 PMC4927921 27376128

[b21] Jourquin J., Duncan D., Shi Z. & Zhang B. GLAD4U: deriving and prioritizing gene lists from PubMed literature. BMC Genomics 13 Suppl 8, S20 (2012). 23282288 10.1186/1471-2164-13-S8-S20 PMC3535723

[b22] Petersen T. N., Brunak S., von Heijne G. & Nielsen H. SignalP 4.0: discriminating signal peptides from transmembrane regions. Nat Methods 8, 785–786 (2011). 21959131 10.1038/nmeth.1701

[b23] Dimmer E. C. . The UniProt-GO Annotation database in 2011. Nucleic Acids Res 40, D565–570 (2012). 22123736 10.1093/nar/gkr1048 PMC3245010

[b24] UniProt C. UniProt: a hub for protein information. Nucleic Acids Res 43, D204–212 (2015). 25348405 10.1093/nar/gku989 PMC4384041

[b25] Gaudet P. . The neXtProt knowledgebase on human proteins: current status. Nucleic Acids Res 43, D764–770 (2015). 25593349 10.1093/nar/gku1178 PMC4383972

[b26] Sarkar P., Randall S. M., Muddiman D. C. & Rao B. M. Targeted proteomics of the secretory pathway reveals the secretome of mouse embryonic fibroblasts and human embryonic stem cells. Mol Cell Proteomics 11, 1829–1839 (2012). 22984290 10.1074/mcp.M112.020503 PMC3518120

[b27] Latosinska A., Frantzi M., Mullen W., Vlahou A. & Makridakis M. Targeting the proteome of cellular fractions: focus on secreted proteins. Methods Mol Biol 1243, 29–41 (2015). 25384738 10.1007/978-1-4939-1872-0_2

[b28] Kulasingam V. & Diamandis E. P. Proteomics analysis of conditioned media from three breast cancer cell lines: a mine for biomarkers and therapeutic targets. Mol Cell Proteomics 6, 1997–2011 (2007). 17656355 10.1074/mcp.M600465-MCP200

[b29] Sardana G., Marshall J. & Diamandis E. P. Discovery of candidate tumor markers for prostate cancer via proteomic analysis of cell culture-conditioned medium. Clin Chem 53, 429–437 (2007). 17259234 10.1373/clinchem.2006.077370

[b30] Grau L. . A quantitative proteomic analysis uncovers the relevance of CUL3 in bladder cancer aggressiveness. PLoS One 8, e53328 (2013). 23308193 10.1371/journal.pone.0053328 PMC3540081

[b31] Makridakis M. . Chromosomal and proteome analysis of a new T24-based cell line model for aggressive bladder cancer. Proteomics 9, 287–298 (2009). 19105184 10.1002/pmic.200800121

[b32] Memon A. A., Chang J. W., Oh B. R. & Yoo Y. J. Identification of differentially expressed proteins during human urinary bladder cancer progression. Cancer Detect Prev 29, 249–255 (2005). 15936593 10.1016/j.cdp.2005.01.002

[b33] Bryan R. T. . Protein shedding in urothelial bladder cancer: prognostic implications of soluble urinary EGFR and EpCAM. Br J Cancer 112, 1052–1058 (2015). 25719831 10.1038/bjc.2015.21 PMC4366887

[b34] Jeppesen D. K. . Quantitative proteomics of fractionated membrane and lumen exosome proteins from isogenic metastatic and nonmetastatic bladder cancer cells reveal differential expression of EMT factors. Proteomics 14, 699–712 (2014). 24376083 10.1002/pmic.201300452

[b35] Makridakis M. . Analysis of secreted proteins for the study of bladder cancer cell aggressiveness. J Proteome Res 9, 3243–3259 (2010). 20423150 10.1021/pr100189d

[b36] Gronborg M. . Biomarker discovery from pancreatic cancer secretome using a differential proteomic approach. Mol Cell Proteomics 5, 157–171 (2006). 16215274 10.1074/mcp.M500178-MCP200

[b37] Kumar B. . p38 mitogen-activated protein kinase-driven MAPKAPK2 regulates invasion of bladder cancer by modulation of MMP-2 and MMP-9 activity. Cancer Res 70, 832–841 (2010). 20068172 10.1158/0008-5472.CAN-09-2918

[b38] Miyata Y. . Lymphangiogenesis and angiogenesis in bladder cancer: prognostic implications and regulation by vascular endothelial growth factors-A, -C, and -D. Clin Cancer Res 12, 800–806 (2006). 16467091 10.1158/1078-0432.CCR-05-1284

[b39] Deng C. . Angiogenic effect of intercellular adhesion molecule-1. J Huazhong Univ Sci Technolog Med Sci 27, 9–12 (2007). 17393097 10.1007/s11596-007-0103-4

[b40] Raman D., Sai J., Neel N. F., Chew C. S. & Richmond A. LIM and SH3 protein-1 modulates CXCR2-mediated cell migration. PLoS One 5, e10050 (2010). 20419088 10.1371/journal.pone.0010050 PMC2856662

[b41] Krause M., Dent E. W., Bear J. E., Loureiro J. J. & Gertler F. B. Ena/VASP proteins: regulators of the actin cytoskeleton and cell migration. Annu Rev Cell Dev Biol 19, 541–564 (2003). 14570581 10.1146/annurev.cellbio.19.050103.103356

[b42] Gomez-Cambronero J. Phospholipase D in cell signaling: from a myriad of cell functions to cancer growth and metastasis. J Biol Chem 289, 22557–22566 (2014). 24990944 10.1074/jbc.R114.574152 PMC4132763

[b43] Shi M., Zheng Y., Garcia A., Xu L. & Foster D. A. Phospholipase D provides a survival signal in human cancer cells with activated H-Ras or K-Ras. Cancer Lett 258, 268–275 (2007). 17949898 10.1016/j.canlet.2007.09.003 PMC3618667

[b44] Valli A. . Hypoxia induces a lipogenic cancer cell phenotype via HIF1alpha-dependent and -independent pathways. Oncotarget 6, 1920–1941 (2015). 25605240 10.18632/oncotarget.3058 PMC4385826

[b45] O’Hayre M., Degese M. S. & Gutkind J. S. Novel insights into G protein and G protein-coupled receptor signaling in cancer. Curr Opin Cell Biol 27, 126–135 (2014). 24508914 10.1016/j.ceb.2014.01.005 PMC4021379

[b46] Dorsam R. T. & Gutkind J. S. G-protein-coupled receptors and cancer. Nat Rev Cancer 7, 79–94 (2007). 17251915 10.1038/nrc2069

[b47] Waugh D. J. & Wilson C. The interleukin-8 pathway in cancer. Clin Cancer Res 14, 6735–6741 (2008). 18980965 10.1158/1078-0432.CCR-07-4843

[b48] Wisniewski J. R., Zougman A., Nagaraj N. & Mann M. Universal sample preparation method for proteome analysis. Nat Methods 6, 359–362 (2009). 19377485 10.1038/nmeth.1322

[b49] Latosinska A. . Comparative Analysis of Label-Free and 8-Plex iTRAQ Approach for Quantitative Tissue Proteomic Analysis. PLoS One 10, e0137048 (2015). 26331617 10.1371/journal.pone.0137048 PMC4557910

[b50] Bairoch A. & Apweiler R. The SWISS-PROT protein sequence database and its supplement TrEMBL in 2000. Nucleic Acids Res 28, 45–48 (2000). 10592178 10.1093/nar/28.1.45 PMC102476

[b51] Eng J. K., McCormack A. L. & Yates J. R. An approach to correlate tandem mass spectral data of peptides with amino acid sequences in a protein database. J Am Soc Mass Spectrom 5, 976–989 (1994). 24226387 10.1016/1044-0305(94)80016-2

[b52] Kall L., Canterbury J. D., Weston J., Noble W. S. & MacCoss M. J. Semi-supervised learning for peptide identification from shotgun proteomics datasets. Nat Methods 4, 923–925 (2007). 17952086 10.1038/nmeth1113

[b53] Shin J. . Use of composite protein database including search result sequences for mass spectrometric analysis of cell secretome. PLoS One 10, e0121692 (2015). 25822838 10.1371/journal.pone.0121692 PMC4378925

[b54] Kim D. . TopHat2: accurate alignment of transcriptomes in the presence of insertions, deletions and gene fusions. Genome Biol 14, R36 (2013). 23618408 10.1186/gb-2013-14-4-r36 PMC4053844

[b55] Cunningham F. . Ensembl 2015. Nucleic Acids Res 43, D662–669 (2015). 25352552 10.1093/nar/gku1010 PMC4383879

[b56] Garcia-Alcalde F. . Qualimap: evaluating next-generation sequencing alignment data. Bioinformatics 28, 2678–2679 (2012). 22914218 10.1093/bioinformatics/bts503

[b57] Anders S., Pyl P. T. & Huber W. HTSeq–a Python framework to work with high-throughput sequencing data. Bioinformatics 31, 166–169 (2015). 25260700 10.1093/bioinformatics/btu638 PMC4287950

[b58] Love M. I., Huber W. & Anders S. Moderated estimation of fold change and dispersion for RNA-seq data with DESeq2. Genome Biol 15, 550 (2014). 25516281 10.1186/s13059-014-0550-8 PMC4302049

[b59] Core Team. R. R: A language and environment for statistical computing. R Foundation for statistical Computing, Vienna, Austria. URL http://www.R-project.org/ (2015).

[b60] Zoidakis J. . Profilin 1 is a potential biomarker for bladder cancer aggressiveness. Mol Cell Proteomics 11, M111 009449 (2012). 22159600 10.1074/mcp.M111.009449 PMC3322560

