# Supplementary material for: Integrative analysis of extracellular and intracellular bladder cancer cell line proteome with transcriptome: improving coverage and validity of –omics findings
Source: Sci Rep. 2016 May 11;6:25619. doi: 10.1038/srep25619 (PMC4863247; doi:10.1038/srep25619)
Supplement: Supplementary Information [file srep25619-s1.pdf]

Integrative analysis of extracellular and intracellular bladder cancer cell line proteome with transcriptome: improving coverage and validity of –omics findings

Agnieszka Latosinska[1,2], Manousos Makridakis[1], Maria Frantzi[3], Daniel M. Borràs[4,5,6], Bart Janssen[4], William Mullen[7], Jerome Zoidakis[1], Axel S. Merseburger[8,9], Vera Jankowski[10], Harald Mischak[3,7], Antonia Vlahou[1]*

[1]Biotechnology Division, Biomedical Research Foundation of the Academy of Athens, Athens, Greece

[2]Charité-Universitätsmedizin Berlin, Berlin, Germany

[3]Mosaiques Diagnostics GmbH, Hannover, Germany

[4]GenomeScan B.V., Leiden, The Netherlands

[5]Institut National de la Santé et de la Recherche Médicale (INSERM), Institut of Cardiovascular and Metabolic Disease, Toulouse, France

[6]Université Toulouse III Paul-Sabatier, Toulouse, France

[7]BHF Glasgow Cardiovascular Research Centre, University of Glasgow, Glasgow, United Kingdom

[8]Department of Urology, University of Lübeck, Lübeck, Germany

[9]Department of Urology and Urological Oncology, Hannover Medical School, Hannover, Germany

[10]RWTH-Aachen, Institute for Molecular Cardiovascular Research (IMCAR), Aachen, Germany

***Corresponding author**

Dr Antonia Vlahou

Biomedical Research Foundation of Academy of Athens

Soranou Ephessiou 4, 115 27 Athens, Greece

Telephone: +30 210 6597 506

Fax: +30 210 6597 545

vlahoua@bioacademy.gr

Supplementary Fig. S1.Reproducibility of collected proteomics data. Graphical representation of number of overlapping proteins among replicates (a) and their contribution to total relative protein abundance (b).

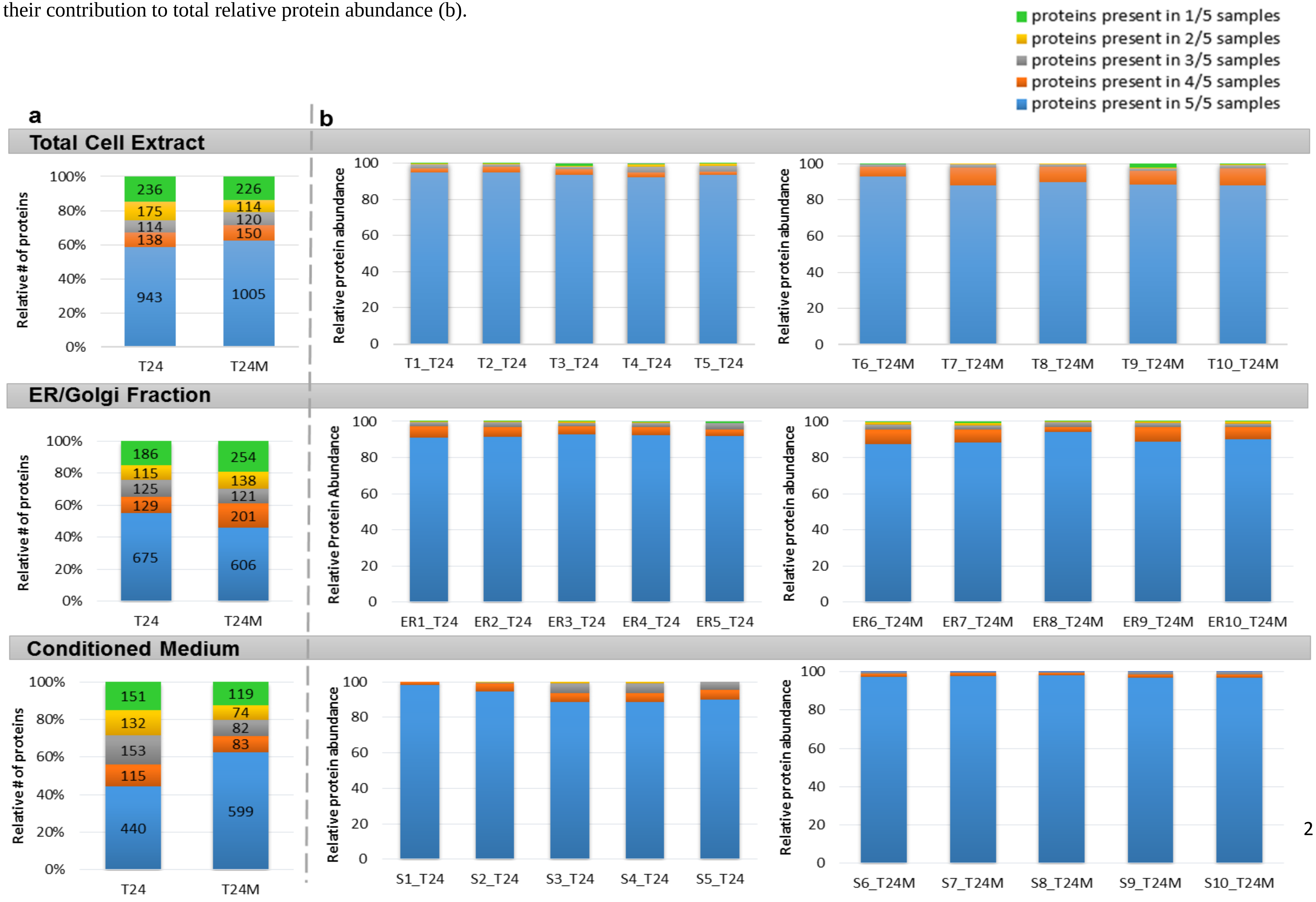

Supplementary Fig. S2. Evaluation of enrichment efficiency of each preparation method. Normalized average intensity values of specific “protein markers” for each preparation method. Actin, cytoplasmic 1 (ACTB), histone H2B type 1-K (HIST1K2BK) (protein markers for CE, a-b); 78kDa glucose regulated protein (HSPA5), calumenin (CALU) (protein markers for ER/Golgi, c-d); Proactivator polypeptide (PSAP) Cathepsin B (CATB) (protein markers for CM, e-f) are shown. Statistical analysis was performed using Mann Whitney Test. For this purpose, intensities corresponding to all samples per preparation (n=10) were pooled.

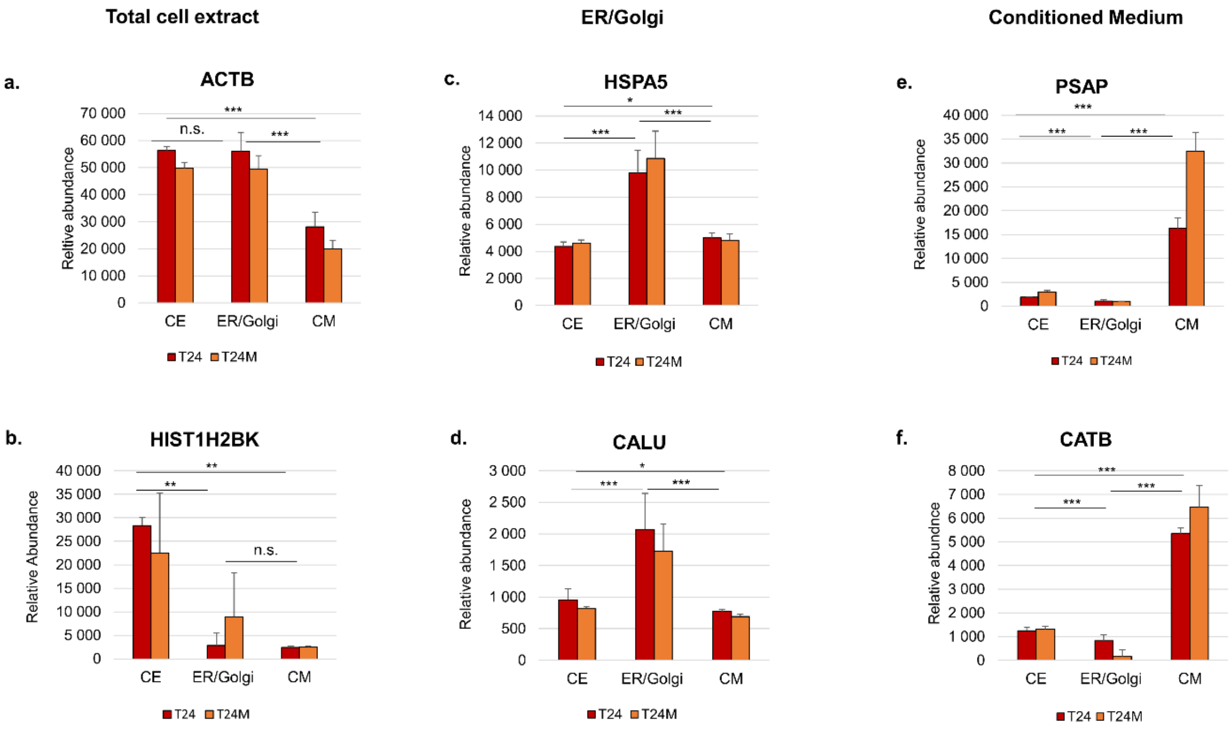

Supplementary Fig. S3. Western blot analysis for VASP protein, supporting decreased levels of the protein in invasive cancers according to the proteomics analysis of ER/Golgi fraction. Western blot analysis of VASP in bladder cancer tissue biopsies (a). Equal loading was assessed by blotting against β-actin (b). Western blot analysis of bladder cancer tissue biopsies support the down-regulation of VASP in muscle-invasive (pT2+) vs. non-muscle invasive bladder cancer (pTa), based on the normalization utilizing beta-actin intensity (c). The same observation was made when testing an additional set of samples (d) in relation to the respective total protein load to the gel (e).

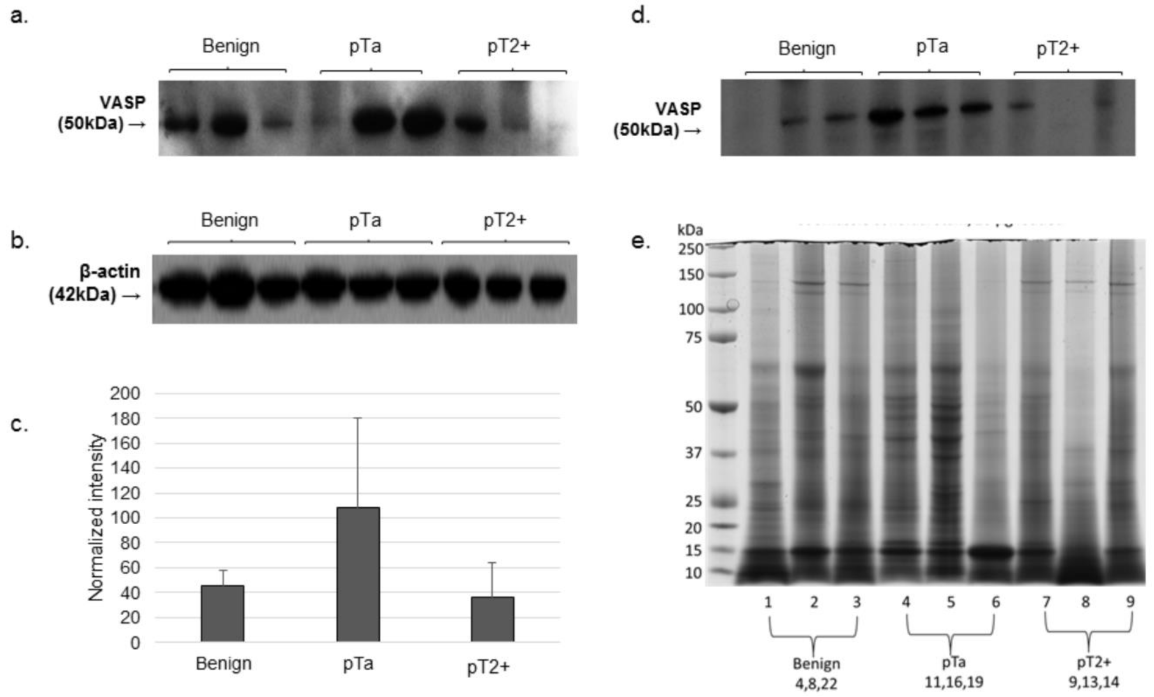

Supplementary Table S1. Proteins identified in individual samples among the three applied experimental approaches (analysis of conditioned medium, ER/Golgi fraction and total cell extract). Proteins identified based on the single peptide as well as proteins with at least 2 unique peptides are presented.

Sample ID	Condtioned medium			ER/Golgi fraction			Total Cell Extract		
	≥ 1 peptide	≥ 2 peptides	Single peptide hits	≥ 1 peptide	≥ 2 peptides	Single peptide hits	≥ 1 peptide	≥ 2 peptides	Single peptide hits
1	807	481	326	1,578	963	615	1,754	1,159	595
2	896	575	321	1,559	942	617	1,959	1,280	679
3	1,274	834	440	1,522	918	604	2,038	1,283	755
4	1,311	889	422	1,551	956	595	1,889	1,213	676
5	1,127	755	372	1,439	908	531	1,972	1,263	709
**Avg. T24 (±SD)**	**1,083 ± 224**	**707 ± 173**	**376 ± 54**	**1,530 ± 55**	**937 ± 24**	**592 ± 35**	**1,922 ± 108**	**1,240 ± 53**	**683 ± 58**
6	1,145	757	388	1,574	1,017	557	2,041	1,314	727
7	1,107	731	376	1,481	945	536	1,960	1,288	672
8	1,050	688	362	1,342	831	511	1,984	1,295	689
9	1,282	865	417	1,562	984	578	1,882	1,244	638
10	1,165	799	366	1,545	957	588	1,958	1,306	652
**Avg. T24M (±SD)**	**1,150 ± 86**	**768 ± 68**	**382 ± 22**	**1,501 ± 96**	**947 ± 70**	**554 ± 31**	**1,965 ± 57**	**1,289 ± 27**	**676 ± 35**
**Avg. T24 & T24M (±SD)**	**1,116 ± 164**	**737 ± 128**	**379 ± 39**	**1,515 ± 75**	**942 ± 50**	**573 ± 37**	**1,944 ± 85**	**1,265 ± 48**	**679 ±45**

Supplementary Table S2. List of the proteins defined as possible contaminants in proteomics mass spectrometry data. The contaminants are originated from the FBS or mass spectrometry reagents.

Conditioned Medium	ER-Golgi	Cell Extract
Alpha-2-macroglobulin<br>Alpha-2-HS-glycoprotein<br>Serum albumin<br>Alpha-fetoprotein<br>Vitamin D-binding protein<br>Inter-alpha-trypsin inhibitor heavy chain H2<br>Inter-alpha-trypsin inhibitor heavy chain H2<br>Hemoglobin subunit alpha<br>Lactotransferrin<br>Trypsin – 1<br>Trypsin – 2	Serum albumin	Serum albumin
